# A cluster of the first reported *Plasmodium ovale* spp. infections in Peru occuring among returning UN peace-keepers, a review of epidemiology, prevention and diagnostic challenges in nonendemic regions

**DOI:** 10.1186/s12936-019-2809-8

**Published:** 2019-05-22

**Authors:** Rosio I. Guerra, Marianela Ore, Hugo O. Valdivia, Danett K. Bishop, Mariana Ramos, Christopher N. Mores, Wesley R. Campbell

**Affiliations:** 1 Dirección de Salud de la Marina, Lima, Peru; 2 Comando de Salud del Ejército, Lima, Peru; 3 U.S. Naval Medical Research Unit No. 6, Lima, Peru; 4 Present Address: Department of Global Health, Milken Institute School of Public Health, The George Washington University, Washington, DC USA; 5 Present Address: Division of Infectious Diseases, Department of Internal Medicine, Walter Reed National Military Medical Center, Bethesda, MD USA

**Keywords:** *Plasmodium ovale*, Relapse, Traveler, Malaria, Non-falciparum malaria

## Abstract

**Background:**

*Plasmodium ovale curtisi* and *Plasmodium ovale wallikeri* are regarded as less virulent forms of malaria with a geographic distribution including Southeast Asia, Central and West Africa, and is increasingly reported as an infection in returning travellers. A species of malaria that may have delayed or relapsing presentations similar to *Plasmodium vivax*, the clinical presentation of *P. ovale* spp. has been described to have prepatent periods of 2 weeks or slightly longer with reports of relapse following primary infection out to 8–9 months. This presentation may be obscured further in the setting of anti-malarial exposure, with report of delayed primary infection out to 4 years. Presented is a cluster of 4 imported *P. ovale* spp. cases in returning Peruvian military personnel assigned to United Nations peace-keeping operations in the Central African Republic.

**Case presentation:**

From January to December 2016, Peruvian peace-keepers were deployed in support of United Nations (UN) operations in the Central African Republic (CAR). While serving abroad, Navy, Army, and Air Force members experienced 223 episodes of *Plasmodium falciparum* malaria following interruption of prophylaxis with mefloquine. Diagnosis was made using rapid diagnostics tests (RDTs) and/or smear with no coinfections identified. Cases of malaria were treated with locally-procured artemether-lumefantrine. Returning to Peru in January 2017, 200 peace-keepers were screened via thick and thin smear while on weekly mefloquine prophylaxis with only 1 showing nucleic acid within red blood cells consistent with *Plasmodium* spp. and 11 reporting syndromes of ill-defined somatic complaints. Between a period of 5 days to 11 months post return, 4 cases of *P. ovale* spp. were diagnosed using smear and polymerase chain reaction (PCR) following febrile complaints. All cases were subsequently treated with chloroquine and primaquine, with cure of clinical disease and documented clearance of parasitaemia.

**Conclusion:**

These patients represent the first imported cases in Peru of this species of malaria as well as highlight the challenges in implementing population level prophylaxis in a deployed environment, and the steps for timely diagnosis and management in a non-endemic region where risk of introduction for local transmission exists.

## Background

*Plasmodium ovale curtisi* and *P. ovale wallikeri* are two non-falciparum malaria (NFM) disease-causing sympatric species endemic to areas in Southeast Asia and particularly in Central and West Africa [1, 2]. A recent systematic review of *P. ovale* spp. infection describes only 18 cases of reported relapse of *P. ovale* spp., 22 reported serious cases, and 5 related deaths in the published literature [3]. Severe cases and mortality are rare, with symptoms of fever occurring when parasitaemia exceeds 800 parasites/µl in populations with endemic circulation [3–6]. Imported cases of this species outside of endemic regions are rare, and the true burden of *P. ovale* spp. disease among residents of endemic areas and among international travellers remains incompletely described.

Reported here are the first imported cases of *P. ovale* spp. in Peru presenting 5 days to 11 months after departure from the Central African Republic (CAR). These cases and the circumstances surrounding the clinical presentation demonstrate the known relapse window for *P. ovale* spp. and highlights the difficulties inherent to assessing risk of transmission to large groups of travellers when making prophylaxis decisions, and the role of diagnostic modalities upon return to permit timely diagnosis and definitive management.

## Case presentation

From January to December 2016, Peruvian forces deployed for 12 months in support of United Nations (UN) peace-keeping operations in the CAR. These forces experienced an outbreak of 223 episodes of malaria attributed to *Plasmodium falciparum* following an interruption in mefloquine prophylaxis. The diagnoses were made in symptomatic individuals using microscopy and locally procured rapid diagnostic tests (RDTs); there were no identified coinfections, and cases were treated with artemether-lumefantrine. In December 2016, prior to return to Peru, deployed personnel received a final month of prophylaxis with weekly dosed mefloquine.

In January 2017, 200 Peruvian peacekeepers underwent thick and thin smear assessment in Lima. Nucleic acid staining within red blood cells was visualized in only one smear. Among the tested personnel, 11 reported non-specific febrile symptoms, with an additional 4 complaining of headache without fever. Personnel were recommended to delay returning to their normal duties at outposts in the Amazon basin while close clinical follow-up and evaluation could be conducted.

**Fig. 1:**
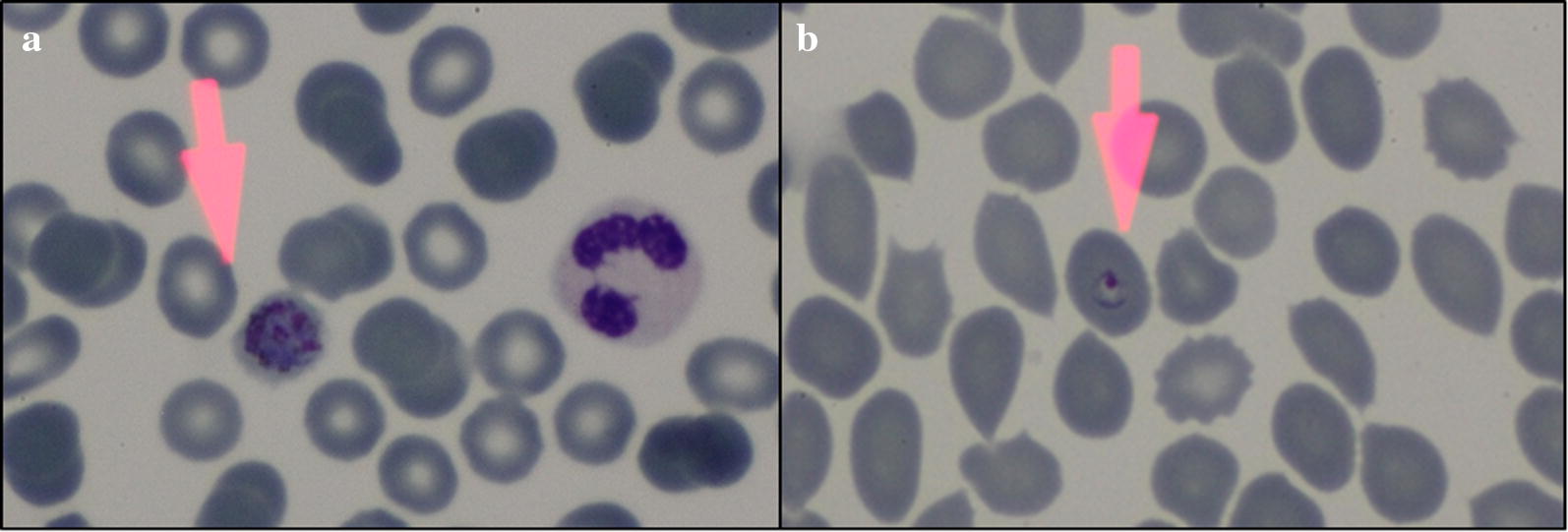
Giemsa thin smear of the PCR confirmed cases: **a** MIS2709 sample (patient 1), ×100 schizont with an estimated 10 merozoites with fimbriation, normal to slightly enlarged red blood cell, consistent with *P. ovale* spp. **b** MIS2712 sample (patient 2), ×100 trophozoite with large chromatin dot

In April 2017, one of the peacekeepers who originally presented with vague complaints and who remained in Lima was diagnosed with *P. ovale* spp. infection on microscopy (Fig. 1a) with a parasitaemia of 778 parasites/µl. He was treated with chloroquine 250 mg daily for 3 days and provided primaquine radical cure dosed at 30 mg (base) daily for 14 days. In November 2017, an additional 3 cases were identified after complaints of fever prompted assessment with microscopy (Table 1). All 4 cases responded to therapy with resolution of symptoms and no evidence of recrudescent malaria on follow-up microscopy.

**Table 1:** *Plasmodium ovale* spp. cases

Patient	Service	Age	Episodes *P. falciparum* (+) in CAR	Primary treatment regimen	Delay in presentation (months)	Presenting complaint	Lab abnormalities	Parasitemia (parasites/µl)	Diagnosis	Treatment regimen
1^a^	Navy	44	1	Artemether 80 mg lumefantrine 480 mg	< 1	BP; (F, HA, GF)^a^	None	778	PCR microscopy	Chloroquine 250 mg primaquine 30 mg (14 days)
2^b^	Army	38	1	Artemether 80 mg lumefantrine 480 mg	11	F, HA, M, A	Th, Tr, IHB	27,339	PCR microscopy	Chloroquine 250 mg primaquine 30 mg (7 days)
3	Army	44	1	Artemether 80 mg lumefantrine 480 mg	11	F, HA, M	Th, Tr, IHB	433	Microscopy	Chloroquine 250 mg primaquine 30 mg (7 days)
4	Army	50	2	Artemether 80 mg lumefantrine 480 mg	11	F, HA, GF, T	Tr	481	Microscopy	Chloroquine 250 mg primaquine 30 mg (7 days)

Delayed presentation time in months from date of return to Peru to seeking care

*CAR* Central Africa Republic, *PCR* polymerase chain reaction, *BP* back pain, *F* fever, *HA* headache, *M* myalgia, *A* arthralgia, *GF* general fatigue, *T* thoracic pain, *Th* thrombocytopenia, *Tr* transaminitis, *IHB* indirect hyperbilirubinemia

^a^Patient with *P. ovale. curtisi*, originally presented with BP 5 days after returning from CAR, symptoms then continued intermittently until April 2017 when diagnosis was made after 2 weeks HA, at 3½ months was admitted with F, BP, M

^b^Patient with *P. ovale. wallikeri* (MIS2712)

**Fig. 2:**
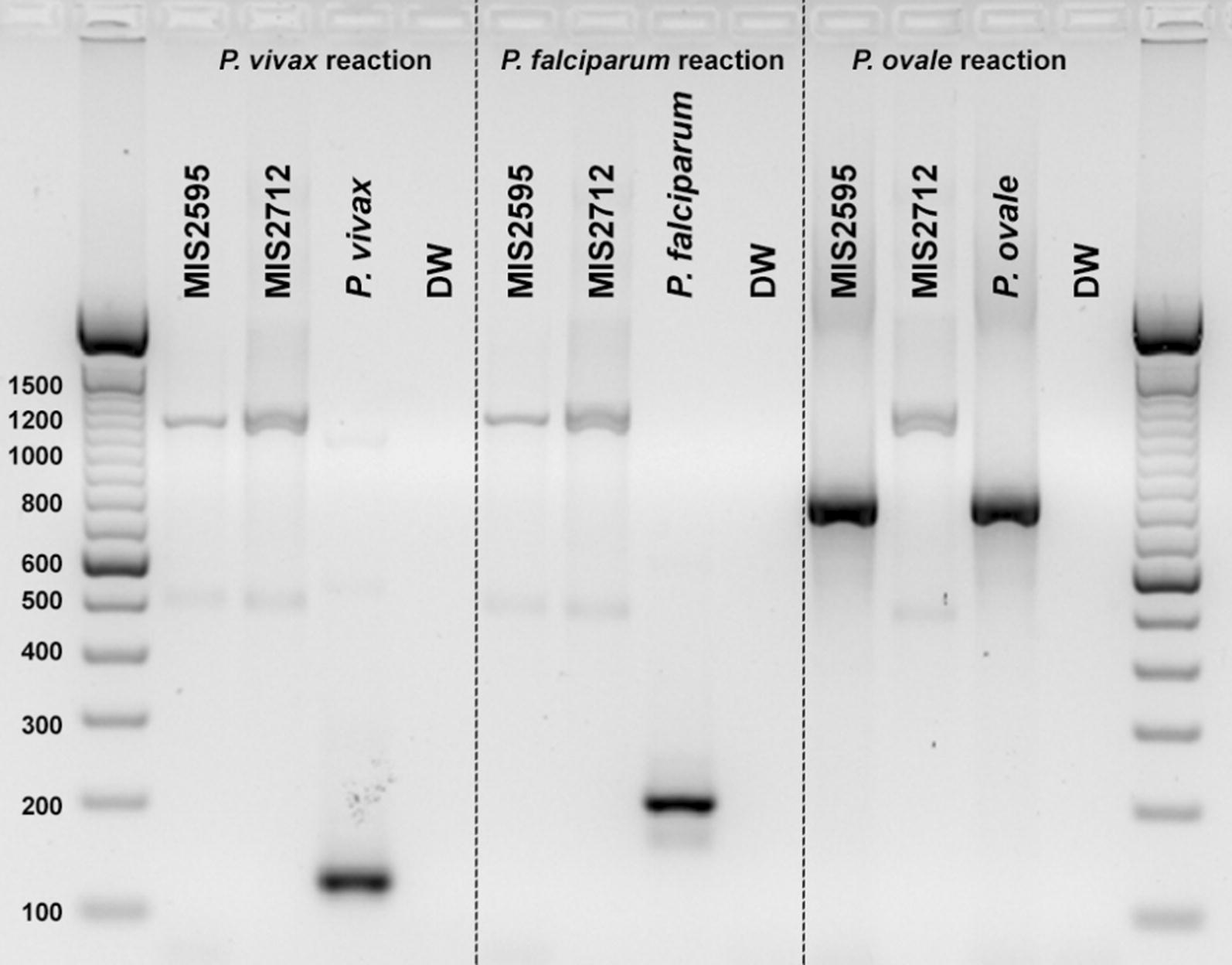
The figure shows the nested species-specific PCR for *P. ovale*. Lanes 1–4 correspond to the *P. vivax* reaction, lanes 5–8 correspond to the *P. falciparum* reaction and lanes 9–12 correspond to the *P. ovale* specific reaction. *P. ovale* was detected on MIS2595, whereas MIS2712 was only positive for *Plasmodium* spp. (≈ 1200 bp). We later determined via sequencing that MIS2712 was *P. ovale wallikeri* which is not detected by the PCR method we employed here

**Fig. 3:**
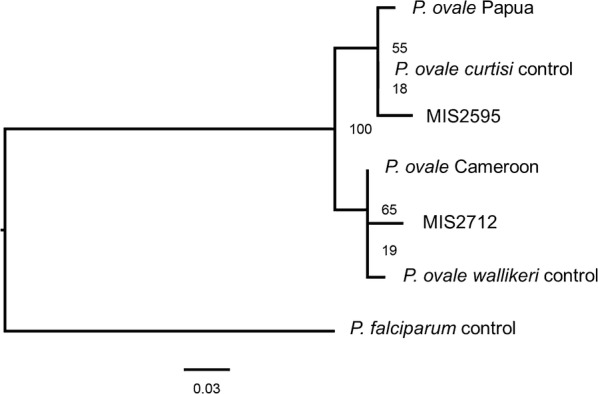
Maximum likelihood phylogenetic tree of the small subunit ribosomal RNA sequence. The tree shows that MIS2595 is phylogenetically related to *P. ovale curtisi* strains whereas MIS2712 is related to *P. ovale wallikeri*. The tree was rooted with the *P. falciparum* 3D7 sequence which show 100% bootstrap support for the *P. ovale curtisi* and *P. ovale wallikeri* nodes

DNA was successfully extracted using established methods from two out of the four cases and used for molecular testing [7, 8]. *Plasmodium ovale* spp. was confirmed for one of the samples by a ≈ 787 bp species-specific band whereas another sample returned positive for *Plasmodium* spp. by a ≈ 1200 bp genus-specific band (Fig. 2). In order to verify the PCR results and confirm the species in the genus positive sample, sequence analysis on the genus PCR product was completed (Fig. 2). The resulting maximum likelihood phylogenetic tree showed that one sample (MIS2595) was phylogenetically related to *P. ovale curtisi* whereas the other (MIS2712) was related to *P. ovale wallikeri* (Fig. 3) [9]. These results were further confirmed following this initial analysis using *P. ovale* spp. specific PCR [10].

## Discussion and conclusions

*Plasmodium ovale* spp. has historically been described as endemic to sub-Saharan and West Africa and Asia and clinically as a less severe form of malaria with lower parasite density [1]. With a prepatent period estimated at 15 days, a reported delayed primary infection presentation on the order of years, and possessing the ability for relapse, this species of malaria can be cause for difficult diagnosis and management for larger groups who travel and for clinicians in non-endemic regions [3, 11]. The context behind this cluster of cases raises these and other questions including just how much of a contribution this species makes to reported imported cases of malaria in South America, how best to prevent infection with prophylaxis, and ultimately is there a risk for introduction of this species and autochthonous spread in the correct setting.

The gold standard for diagnosis and correlation of parasitaemia to presentation remains visualization on microscopy [12]. Although *P. ovale* spp. may be diagnosed with microscopy and species confirmed with PCR, *P. ovale* spp. may be confused for *Plasmodium vivax*, making the diagnostic approach balanced to the resources available, the patient or population affected, and the expertise of the lab assisting in confirming diagnosis [13–16]. RDTs are less sensitive for detection and rely on aldolase or pLDH antigen to detect NFM. Performance of RDTs for NFM is consistently below 90% sensitivity, with correct identification ranging between 28.3 and 35.6% [16, 17]. Overall, the sensitivity of available diagnostic tests for NFM is noted to be lowest for RDTs, increased with microscopy, and the highest with PCR [17].

The role of PCR in regions where a species may be non-endemic has been recognized as a valuable tool for confirming or making a diagnosis. One reference laboratory-based study in Israel reported a low rate of 11% correct diagnosis for *P. ovale* spp. with smear or RDTs and demonstrated the value of PCR technology [18]. In a similar population to these cases, universal PCR screening has been instituted by Guatemalan forces following a documented case of *P. ovale* spp. in a returning UN peace-keeper [13]. The detection limits of *P. ovale* spp. PCR can be as low as 1 parasite/µl of blood depending on the target and type PCR of method, and despite being time consuming is useful in aiding diagnosis in smear or RDT negative cases [18, 19]. When used as a tool in epidemiological evaluation for control, PCR as a diagnostic tool has been critical when exploring the suspected reemergence of *P. vivax* in an area of prior eradication, only to be identified as *Plasmodium simium* cases [20].

Treatment of *P. ovale* spp. is largely based on experience from *P. vivax*, with the role of primaquine in radical cure not as elucidated for this species. The mechanism of *P. ovale* spp. relapse is a matter of recent debate in the literature, and the parallels with *P. vivax* with regard to latent sporozoite activation or a form of merozoite development having not been clearly described [21]. Periods between primary infection and periods of clinical illness or relapse may be on the order of years and add to the clinical complexity of making the diagnosis [11]. Treatment is further complicated by uncertainty regarding the dosing of primaquine for *P. ovale* spp., with variation in practice based on regional experience and courses ranging from 7 to 14 days, weight-based recommendations ranging from 0.25 to 0.5 mg of base/kg/day, and dosing dependent on interpretation of base for salt components [22, 23]. Peruvian treatment experience and guidelines for *P. vivax* support a 7-day course of primaquine dosed at 0.5 mg base/kg/day [24]. These cases were managed as described in Table 1, with primaquine dosing and course at 7–14 days, matching recommendations of either U.S. Centers for Disease Control (CDC) and local Peruvian guidelines for *P. vivax* [25].

The contribution of *P. ovale* spp. to the burden of malaria in Africa is estimated as high as 10%, commonly in the background of mixed infection with *P. falciparum*, to as low as 2.5% in asymptomatic cross-sectional assessments in one 20-year longitudinal study [4]. Concerning the risk of *P. ovale* spp. emerging following or being missed by control measures for *P. falciparum,* Roucher et al. [4] demonstrated a near-absence of *P. ovale* spp. following the introduction of general malaria control measures when switching from chloroquine to amodiaquine and sulfadoxine/pyrimethamine in Senegal. The absence of the Duffy antigen group in South and West African populations, considered to be somewhat protective for *P. vivax* infection and modulate clinically apparent disease based on recent evidence [26–28], is not protective for infection with *P. ovale* spp. [1]. Evidence suggests that *P. ovale* spp. inoculation induces the rapid acquisition of immunity and that asymptomatic relapses are likely to occur [4–6] True relapses have been rarely reported, making delayed primary presentation in the setting of exposure to anti-malarials a likely scenario for our cluster of cases [1, 3].

Peruvian personnel have supported international UN peacekeeping missions since 2004, with deployments to Haiti as well as the CAR. Increasingly, *P. ovale* spp. is recognized as a cause of fever due to malaria in returning travellers to areas around the world. Researchers in Spain identified 102 cases imported between 2005 and 2011 in a multi-centre retrospective review, ranging between 2 and 8% of cases in a subgroups of immigrants and travellers [16]. Similarly, in Portugal, 6 confirmed cases of *P. ovale* spp. were identified with a possible 3 additional cases identified as *P. ovale* spp. or *P. vivax* [15]. Additional cases have been described in Latin America, most frequently in the context of travel to Africa and until recently, relied solely upon microscopy [29–31]. Cases in deployed forces returning from Africa with well-described periods of symptom onset or relapse as far out as 5 years have been described, while the U.S. military reported 1 case of *P. ovale* spp. from Afghanistan 2016 [11, 14, 32].

This cluster of cases experienced either a delayed primary presentation or relapse in the context of mefloquine prophylaxis and treatment for *P. falciparum*, a known potential confounder of *P. ovale* spp. presentation [11, 33]. Delay in presentation between the two species in relation to season of exposure and malaria prophylaxis use among travellers from nonendemic regions has been described as a clinical feature of these species [34, 35]. Of 757 cases in a UK database spanning 8 years, 33% used prophylaxis with *P. ovale curtisi* and *P. ovale wallikeri* possessing mean latency periods of 85.7 and 40.6 days, respectively [35]. Of the small subgroup in this retrospective study with *P. ovale wallikeri* and *P. ovale curtisi*, latency appeared to be decreased to 69.9 days and lengthened to 60.4 days respectively when prophylaxis regimens were used. Our patients, presenting between 1 and 11 months (Table 1) and with a presumed acquisition risk transcending the transmission season (August through November), mirror findings of a retrospective UK analysis [34]. In this study 23.4% of presentations were beyond 90 days of latency and that latency may be driven by timing of exposure as it relates to malaria season, 44 days within vs. 94 days outside the peak transmission season and is postulated to be an evolutionary adaptation to improve success of transmission [34]. Mefloquine with a prolonged termination half-life of 13.8–40.9 days, coupled with artemether–lumefantrine treatment may have delayed or altered the presentation of *P. ovale* spp. among these cases [36, 37]. Presumptive anti-relapse therapy with primaquine as part of prophylaxis strategies is recommended when traveling to *P. vivax* and *P. ovale* spp. endemic regions and is recognized as a strategy to prevent relapse in *P. ovale* spp. infections [38]. Nevertheless, its use in travellers or large military units requires providers to forecast risk of acquisition of this species of malaria and consider possible consequences of importation of this NFM to a non-endemic region [38–40].

As regions and countries progress in malaria elimination campaigns, imported cases of malaria become an increasing risk for reestablishing endemicity or introduction of new species [41]. Reintroduction of once eliminated species of malaria has been a noted concern in the literature, centering around cross border migration of populations to areas where competent vectors exist and where the proportion of imported cases is increasing [40, 42]. This is especially a concern during the prevention and reintroduction periods of elimination campaigns where malaria may be near eradication and where competent vectors exist or expanding their geographic distribution to suitable areas of transmission in the era of climate change [43]. These same concerns have been shared by UN peace-keeping forces from Sri Lanka, a country with malaria eradication since 2012, experiencing malaria outbreaks during their time of service in the CAR [44].

This is the first reported instance of imported *P. ovale* spp. to Peru, occurring in the context of international operations. These 4 cases bring to the forefront many of the limitations in the understanding of this NFM; risk to the individual and large groups of travellers, and how best to diagnose and manage these patients. While this particular infecting species may be considered less severe than other *Plasmodium* species, the clinical significance of the subspecies of *P. ovale* spp. is still being described [16]. There is much to be learned from this cluster of *P. ovale* spp. cases as an aetiology of ill-defined fever in large groups of travellers returning to a non-endemic region with the potential for its autochthonous transmission by the local competent vector, *Anopheles darlingi*.

## Data Availability

Available upon reasonable request.

## Abbreviations

NFM: non falciparum malaria
CAR: Central African Republic
UK: United Kingdom
UN: United Nations
RDT: rapid diagnostic test
